# Adult tethered cord syndrome associated with spina bifida occulta and a lipomatous filum terminale: case report and structured literature review

**DOI:** 10.1016/j.bas.2026.106177

**Published:** 2026-07-17

**Authors:** Christopher McGinley, Nuha Mohammed, Damirez T. Fossett, Noor Malik, Riaz Muhammad, Rauf Nasirov

**Affiliations:** a Howard University College of Medicine, 520 W Street NW, DC, Washington, 20059, USA; b Division of Neurosurgery, Howard University Hospital, 2041 Georgia Avenue NW, DC, Washington, 20060, USA; c Division of Neurosurgery, Denver Health Medical Center, 660 N. Bannock St., Pavilion L, 5th Floor, Denver, CO, 80204, USA

**Keywords:** Adult tethered cord syndrome, Filum terminale lipoma, Spina bifida occulta, Neurogenic bladder, Detethering, Occult dysraphism

## Abstract

**Introduction:**

Adult tethered cord syndrome (TCS) is uncommon and may be overlooked when chronic lumbosacral pain coexists with subtle occult dysraphism and urinary complaints.

**Research question:**

How should an adult patient with back pain, recurrent urinary tract infections, and imaging evidence of a low-lying conus with a lipomatous filum terminale be evaluated and managed?

**Material and methods:**

We report a 27-year-old woman with progressive low back and left leg pain, recurrent urinary tract infections, and detrusor overactivity. Computed tomography showed L5-S1 spina bifida occulta, and magnetic resonance imaging showed a low conus ending at L3/4 with a thickened lipomatous filum terminale. She underwent L5 laminectomy and microsurgical detethering with intraoperative neurophysiological monitoring. A structured literature review of adult TCS related to lipomatous filum, occult dysraphism, and urinary dysfunction was also performed.

**Results:**

The filum was thickened and fatty intraoperatively, with no evoked responses up to 1.5 mA before sectioning. After surgery, back and radicular pain resolved, urinary tract infections did not recur, no new neurological deficit developed, and the incision was well healed at 7 weeks. The adult literature shows that pain and urinary dysfunction are common presenting features and that detethering most consistently improves pain.

**Discussion and conclusion:**

Adult TCS should remain in the differential diagnosis for persistent low back pain with urinary dysfunction, even when neurological examination is normal and cutaneous stigmata are absent. In symptomatic adults, a low conus, lipomatous filum terminale, and spina bifida occulta should prompt consideration of detethering.

## Highlights

• Adult tethered cord can present with pain and recurrent urinary symptoms.
• Low conus plus lipomatous filum and occult dysraphism should raise suspicion.
• Microsurgical detethering relieved pain and stopped recurrent UTIs in this case.
• Focused adult literature supports surgery when symptoms match imaging.

## 1 Introduction

Tethered cord syndrome (TCS) is a neurological disorder caused by abnormal traction on the spinal cord due to congenital anomalies or acquired lesions. The overall incidence is estimated at approximately 0.25 per 1000 live births (25 per 100,000), with most cases presenting in childhood rather than adulthood, though advances in neuroimaging and heightened clinical awareness have improved recognition, especially in adults (He et al., 2024).

TCS presentation is highly variable. Pediatric patients often possess overt cutaneous stigmata such as sacral dimples, hypertrichosis, or subcutaneous lipomas. Pediatric patients may present clinically with scoliosis, foot deformities, gait disturbances, and bladder or bowel dysfunction, including constipation (Habib et al., 2025; Shukla et al., 2018).

By contrast, adult-onset or adult-diagnosed TCS is relatively rare, and adults typically lack the external indicators seen in pediatric patients. Instead, magnetic resonance imaging (MRI) may reveal features such as spina bifida occulta, a thickened or fatty filum terminale, intradural lipomas, or a low-lying conus medullaris (Habib et al., 2025). Clinically, adults more often present with chronic back pain, lower-extremity radiculopathy, sensory loss, motor impairment, and urinary dysfunction, including recurrent urinary tract infections (UTIs) (Kanda et al., 2015; Leary et al., 2023).

Surgical detethering remains the gold standard treatment across all age groups, though outcomes vary depending on the timing of intervention and the complexity of the pathology. In adults, outcomes are generally favorable: in one large series of 57 patients, 93% experienced improved or stable muscle strength with subjective improvement in pain, gait, and bladder/bowel function over two years, without major complications (Shukla et al., 2018).

Here, we report the case of an adult woman with chronic back pain and recurrent UTIs, found to have TCS with an intrafilum lipoma and spina bifida occulta, who experienced resolution of symptoms following surgical detethering.

## 2 Materials and methods

### 2.1 Clinical case

A 27-year-old female presented with a 2-year history of progressive low back pain radiating to the left thigh, leg, and foot. Pain intensity was reported as severe (10/10 at worst), exacerbated by physical activity. She also had a history of recurrent UTIs but denied bowel dysfunction or obvious urinary incontinence. No cutaneous lumbosacral stigmata were identified.

Physical and neurological examination were unremarkable, with intact cranial nerve function, full motor strength (5/5) in all extremities, normal sensation, and symmetric deep tendon reflexes. A voiding cystourethrogram revealed detrusor overactivity with a thick-walled bladder. These findings were later confirmed on a CT abdominal scan, which, although performed for other complaints, revealed a thickened urinary bladder wall with associated perilesional fat stranding, consistent with recurrent UTIs.

Spinal imaging revealed structural abnormalities consistent with tethered cord syndrome. CT imaging of the lumbar spine demonstrated spina bifida occulta at L5–S1 with non-fusion of the posterior vertebral elements (Fig. 1). MRI of the lumbosacral spine showed a low-lying conus medullaris terminating at the L3/L4 junction, a thickened filum terminale, and cauda equina nerve roots displaced bilaterally by the tethered cord (Fig. 2). Given her clinical symptoms, radiological findings, and recurrent UTIs with possible neurogenic bladder, surgical detethering was recommended to prevent further neurological and orthopedic sequelae, including scoliosis. A sagittal T1-weighted MRI further demonstrated the lipomatous lesion within the filum terminle and postoperative 3 months follow-up MRI showed its interval removal following detethering (Fig. 3), (see Fig. 4).

**Fig. 1:**
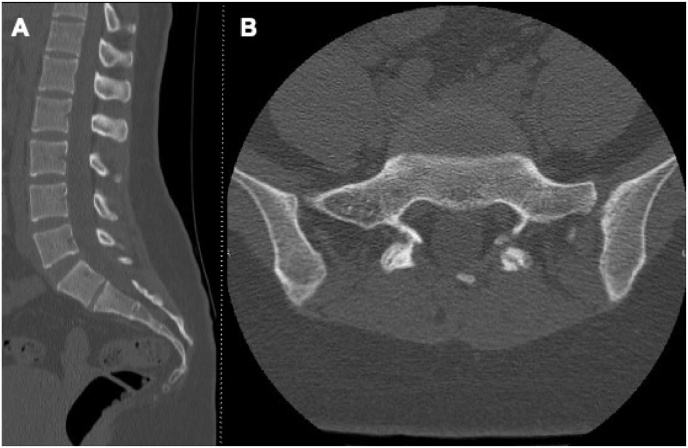
Sagittal (A) and axial (B) bone-window computed tomography images demonstrating spina bifida occulta at L5-S1 with nonfusion of the posterior vertebral elements.

**Fig. 2:**
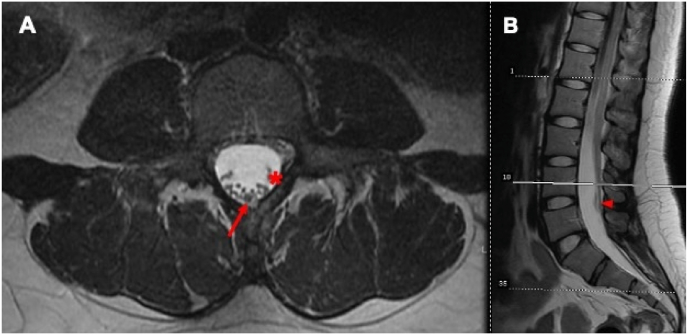
Lumbar magnetic resonance imaging. Axial T2-weighted image (A) demonstrates a thickened filum terminale (arrow) and displaced cauda equina nerve roots (asterisk). Sagittal image (B) shows a low-lying conus medullaris terminating at L3 with tethering and a thickened lipomatous filum terminale (arrowhead).

**Fig. 3:**
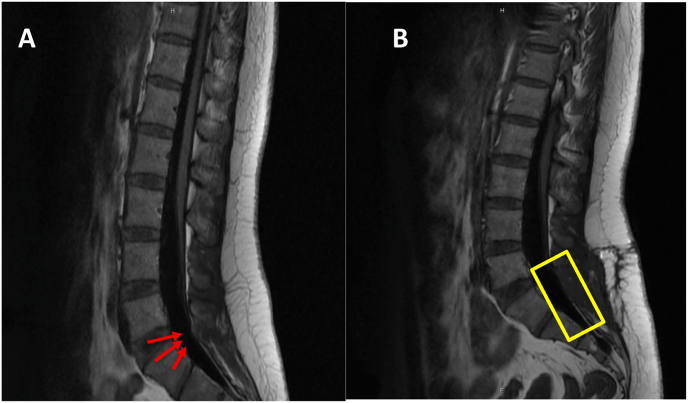
Preoperative (A) T1 sagittal MRI of lumbar spine - red arrows in the preoperative scan indicate the lesion. and (B) three-month postoperative T1 sagittal MRI of lumbar spine - yellow box on postoperative imaging displaying removal of lesion.

**Fig. 4:**
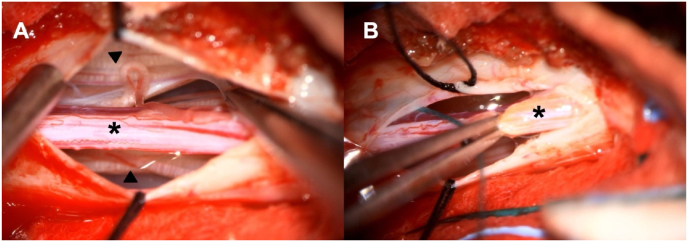
Intraoperative photomicrographs. (A) Thickened filum terminale (asterisk) with adjacent nerve roots (arrowheads). (B) Close-up view showing intrinsic fatty change within the filum (asterisk), consistent with a lipomatous filum terminale.

### 2.2 Structured literature review

A structured literature search was performed in PubMed and Google Scholar using combinations of the terms “adult tethered cord syndrome,” “filum terminale lipoma,” “filar lipoma,” “spina bifida occulta,” “urinary dysfunction,” “neurogenic bladder,” and “detethering.” English-language case reports, case series, reviews, and outcome studies describing adults (18 years or older) with TCS related to a lipomatous or thickened filum and/or occult dysraphism were reviewed. Reference lists of selected articles were screened for additional relevant publications. The purpose of the review was tutorial and case-focused rather than meta-analytic; representative adult studies most relevant to diagnosis, urinary manifestations, and operative outcomes are summarized in Table 1.

**Table 1:** Representative adult literature relevant to the present case.

Ref.	Study focus	Study type/population	Relevance to current case
He et al. (2024)	Systematic review of pediatric and adult TCS	Large review of 6135 cases	Supports the adult phenotype of pain, urinary symptoms, and delayed recognition.
Shukla et al. (2018)	Clinicoradiological differences between adult and pediatric TCS	Comparative series/review	Supports the distinction between adult pain/radiculopathy/bladder dysfunction and pediatric external stigmata.
Kanda et al. (2015)	Unusual adult presentation	Case report	Shows that adult TCS may present atypically, strengthening the diagnostic lesson of the present case.
Leary et al. (2023)	Modern adult interdisciplinary spine series	Adult case series	Provides contemporary adult outcome data after detethering and supports surgery when symptoms and imaging are concordant.
Düz et al. (2008)	Adult TCS outcome analysis	Adult surgical series	Supports the frequency of back pain and bladder dysfunction and documents postoperative improvement.
(Abdulrazeq et al., 2023; Selçuki et al., 2003; Klinge et al., 2022a, 2022b; Pang et al., 2011)	Filum pathology and tethering mechanisms	Histopathology, biomechanics, and developmental studies	Supports the biological plausibility of symptoms arising from an abnormal filum terminale even in subtle radiographic cases.
(Selçuki et al., 2000), (Romagna et al., 2013)	Functional outcomes after detethering	Outcome studies	Support the clinical relevance of urinary symptoms and the role of surgery in symptomatic tethering.

## 3 Results

### 3.1 Operative findings and postoperative course

The patient underwent L5 laminectomy and microsurgical detethering under intraoperative neurophysiological monitoring. Operative inspection confirmed an incomplete left L5 lamina, a low-lying conus, and a thickened filum with intrinsic fatty change consistent with a lipomatous filum terminale (Fig. 3). Electrical stimulation of the filum did not elicit responses up to 1.5 mA, supporting safe sectioning. The filum was divided and the cord was released without complication.

Postoperatively, the patient had complete resolution of low back and radicular pain, no new neurological deficit, and no recurrent UTIs during the available follow-up. At 7 weeks, she remained symptom-free and her incision was well healed.

### 3.2 Findings from the structured literature review

Across the adult-focused literature already cited in the main manuscript, pain, urinary symptoms, and delayed diagnosis emerge as the dominant themes. He et al. found that adults most commonly presented with pain, urinary symptoms, and numbness in a comprehensive review of pediatric and adult cases (He et al., 2024). Shukla et al. similarly emphasized that adults differ from children by presenting more often with chronic back pain, radiculopathy, and bladder dysfunction rather than cutaneous markers or orthopedic deformity (Shukla et al., 2018). Leary et al. and Düz et al. both showed that adult patients frequently come to surgery after prolonged symptoms, often in the setting of otherwise subtle clinical findings (Leary et al., 2023; Düz et al., 2008).

The same reference framework also supports careful attention to imaging and filum pathology. Ultrasonographic and MRI studies have shown measurable differences in filum thickness and improved visualization of filum abnormalities, although imaging does not always perfectly correlate with symptoms (Keykhosravi et al., 2023; Rafiee et al., 2023). Histopathological studies have demonstrated that the filum terminale may harbor abnormal connective tissue and neural elements even when gross appearance is not dramatic, which helps explain why symptomatic tethering can exist in radiographically subtle cases (Abdulrazeq et al., 2023; Selçuki et al., 2003). The concept that the filum is not merely a passive anchor is further supported by studies demonstrating stretch-sensitive and nociceptive nerve endings and altered viscoelastic properties in diseased tissue (Klinge et al., 2022a, 2022b). Developmental abnormalities such as retained medullary cord further broaden the pathological spectrum of tethering (Pang et al., 2011).

Outcome data within the original reference set suggest that detethering most consistently improves pain, whereas urinary improvement is more variable but still clinically important. In the adult case series by Leary et al., neurologic symptoms decreased from 100% to 77.8% and back pain from 87.8% to 48.9% one month after detethering (Leary et al., 2023). Düz et al. reported postoperative improvement rates of 78% for back pain, 83% for leg pain, and 50% for bladder function among symptomatic adults (Düz et al., 2008). Romagna et al. likewise found that adults can improve after detethering, although delayed diagnosis was associated with less complete recovery (Romagna et al., 2013). In addition, Selçuki et al. showed that patients with urinary incontinence often benefit from detethering of a tight filum terminale, reinforcing the clinical importance of bladder symptoms when selecting patients for surgery (Selçuki et al., 2000).

## 4 Discussion

TCS in adults remains diagnostically challenging, as clinical manifestations are often nonspecific and imaging findings may be subtle. The underlying pathophysiology centers on chronic mechanical traction of the spinal cord, which impairs spinal cord blood flow, resulting in ischemia, gliosis, and progressive neurological decline (He et al., 2024; Düz et al., 2008). In our patient, a low-lying conus medullaris and thickened, lipomatous filum terminale provided radiological confirmation of tethering. Additional CT abdominal imaging findings of ureteral wall thickening and perilesional fat stranding were consistent with recurrent UTIs, a common manifestation of sacral parasympathetic involvement in TCS.

Notably, the clinical spectrum of TCS differs between pediatric and adult populations. Children most often present with scoliosis, gait disturbances, cutaneous stigmata, and motor delays, reflecting congenital etiologies and early neurological impairment (Habib et al., 2025; Shukla et al., 2018). By contrast, adults more frequently report chronic back pain, radiculopathy, paresthesia, weakness, and bladder dysfunction, often in the absence of external markers (Habib et al., 2025; Shukla et al., 2018). Rare and atypical cases may occur, as illustrated by Kanda et al., who described sudden-onset chest and upper back pain with subsequent urinary retention in an adult TCS patient (Kanda et al., 2015). These heterogeneous presentations contribute to diagnostic delays, particularly in adults, where the absence of overt congenital markers demands a higher index of clinical suspicion. A comparative overview of clinical features in adult versus pediatric tethered cord syndrome is summarized in Table 2.

**Table 2:** Differences between pediatric and adult tethered cord syndrome.

Features	Pediatric TCS	Adult TCS
**Cause**	Congenital (e.g., myelomeningocele)	Acquired (scar tissue, lipomas) or congenital lesions presenting later
**Presentation**	Motor/sensory deficits, gait abnormalities	Non radicular back pain, bladder dysfunction, recurrent UTIs
**Imaging**	Low-lying conus, thickened filum terminale, and other spinal anomalies such as lipomas.	Similar
**Surgical Outcomes**	High success rate with early intervention	Delayed surgery may reduce effectiveness
**Risk of Retethering**	Yes	Yes

Imaging advances have refined the detection of subtle tethering abnormalities. Ultrasonography, while more frequently used in pediatric populations, has shown measurable differences in filum thickness and spinal canal dynamics in patients with TCS compared to controls (Keykhosravi et al., 2023). Similarly, MRI refinements, including 3D gradient-echo sequences, have improved visualization of filum anomalies (Rafiee et al., 2023). Nonetheless, imaging may not always correlate with symptoms, as some filum terminale samples that appear normal radiographically are shown histologically to harbor pathological changes (Abdulrazeq et al., 2023; Selçuki et al., 2003).

Histopathology has increasingly been recognized as a central component to the pathogenesis of TCS. Large cohort analyses reveal that the filum terminale often contains abnormal connective tissue and nerve fibers, suggesting that it functions as more than a passive anchoring structure (Abdulrazeq et al., 2023; Klinge et al., 2022a). Characteristic findings include reduced elastin, increased collagen density, and altered viscoelastic properties, which may be amplified in systemic connective tissue disorders such as Ehlers–Danlos syndrome (Klinge et al., 2022b). Developmental anomalies also play a role: Pang et al. identified retained medullary cord as an embryologic remnant of defective secondary neurulation, further expanding the spectrum of tethering mechanisms (Pang et al., 2011).

Surgical detethering is widely supported as an effective treatment across both pediatric and adult cases. In children, detethering is associated with significant improvements in constipation, urinary incontinence, and motor function (Habib et al., 2025; Selçuki et al., 2000). In adults, outcomes are favorable but more variable, with improvements often limited by the chronic nature of symptoms and by delayed diagnosis (Leary et al., 2023; Romagna et al., 2013). In a comprehensive interdisciplinary spine center cohort, Leary et al. reported that neurologic symptoms decreased from 100% to 77.8% and back pain from 87.8% to 48.9%, one month after detethering, highlighting a meaningful short-term benefit.5 Romagna et al. similarly reported significant neurological and pain improvements in adults following detethering, though delayed diagnosis correlated with less complete recovery (Romagna et al., 2013). In another large cohort, post-operative improvement rates were high for back pain (78%), leg pain (83%), and bladder function (50% in those with preoperative dysfunction) (Düz et al., 2008).

Prognosis depends on several factors. Primary surgeries for isolated tethered cord typically yield better outcomes than revision procedures or complex malformations (Düz et al., 2008; Abdulrazeq et al., 2023). High cord tension is recognized as a principal pathological driver (He et al., 2024; Klinge et al., 2022a). While early surgery provides the best chance for neurological recovery, late intervention can still alleviate pain and stabilize progression (Shukla et al., 2018; Düz et al., 2008).

## 5 Conclusion

This case contributes to the growing recognition that adult-diagnosed TCS, though less common than pediatric presentations, should remain a critical differential diagnosis in patients with chronic pain, neurological symptoms, or unexplained urinary dysfunction, without cutaneous stigmata or major neurological deficits. Early recognition and surgical management can significantly improve outcomes and prevent irreversible neurological deterioration.

## Patient consent

Written informed consent for publication of the clinical details and images was obtained from the patient.

## Ethics statement

This study complies with the Declaration of Helsinki and principals of Good Clinical Practice.

## Author contributions

Christopher McGinley: conceptualization, data curation, writing-original draft. Nuha Mohammed: literature review, writing-original draft. Damirez T. Fossett: surgery, supervision, writing-review & editing. Noor Malik: surgery, supervision, writing-review & editing. Riaz Muhammad: writing-review & editing. Rauf Nasirov: conceptualization, validation, writing-review & editing. All authors approved the final manuscript.

## Funding

This research did not receive any specific grant from funding agencies in the public, commercial, or not-for-profit sectors.

## Declaration of competing interest

The authors declare that they have no known competing financial interests or personal relationships that could have appeared to influence the work reported in this paper.
