# Supplementary material for: Adult tethered cord syndrome associated with spina bifida occulta and a lipomatous filum terminale: case report and structured literature review
Source: Brain Spine. 2026 Jul 17;6:106177. doi: 10.1016/j.bas.2026.106177 (PMC13396941; doi:10.1016/j.bas.2026.106177)
Supplement: Multimedia component 1 [file mmc1.docx]

**Structured literature review**

A structured literature search was performed in PubMed and Google Scholar using combinations of the terms “adult tethered cord syndrome,” “filum terminale lipoma,” “filar lipoma,” “spina bifida occulta,” “urinary dysfunction,” “neurogenic bladder,” and “detethering.” English-language case reports, case series, reviews, and outcome studies describing adults (18 years or older) with TCS related to a lipomatous or thickened filum and/or occult dysraphism were reviewed. Reference lists of selected articles were screened for additional relevant publications. The purpose of the review was tutorial and case-focused rather than meta-analytic; representative adult studies most relevant to diagnosis, urinary manifestations, and operative outcomes are summarized in Table 1.

**Findings from the structured literature review**

Across the adult-focused literature already cited in the main manuscript, pain, urinary symptoms, and delayed diagnosis emerge as the dominant themes. He et al. found that adults most commonly presented with pain, urinary symptoms, and numbness in a comprehensive review of pediatric and adult cases [1]. Shukla et al. similarly emphasized that adults differ from children by presenting more often with chronic back pain, radiculopathy, and bladder dysfunction rather than cutaneous markers or orthopedic deformity [3]. Leary et al. and Düz et al. both showed that adult patients frequently come to surgery after prolonged symptoms, often in the setting of otherwise subtle clinical findings [5,6].

The same reference framework also supports careful attention to imaging and filum pathology. Ultrasonographic and MRI studies have shown measurable differences in filum thickness and improved visualization of filum abnormalities, although imaging does not always perfectly correlate with symptoms [7,8]. Histopathological studies have demonstrated that the filum terminale may harbor abnormal connective tissue and neural elements even when gross appearance is not dramatic, which helps explain why symptomatic tethering can exist in radiographically subtle cases [9,10]. The concept that the filum is not merely a passive anchor is further supported by studies demonstrating stretch-sensitive and nociceptive nerve endings and altered viscoelastic properties in diseased tissue [11,12]. Developmental abnormalities such as retained medullary cord further broaden the pathological spectrum of tethering [13].

Outcome data within the original reference set suggest that detethering most consistently improves pain, whereas urinary improvement is more variable but still clinically important. In the adult case series by Leary et al., neurologic symptoms decreased from 100% to 77.8% and back pain from 87.8% to 48.9% one month after detethering [5]. Düz et al. reported postoperative improvement rates of 78% for back pain, 83% for leg pain, and 50% for bladder function among symptomatic adults [6]. Romagna et al. likewise found that adults can improve after detethering, although delayed diagnosis was associated with less complete recovery [15]. In addition, Selçuki et al. showed that patients with urinary incontinence often benefit from detethering of a tight filum terminale, reinforcing the clinical importance of bladder symptoms when selecting patients for surgery [14].
